# The recombinant anti-TNF-α fusion protein ameliorates rheumatoid arthritis by the protective role of autophagy

**DOI:** 10.1042/BSR20194515

**Published:** 2020-09-18

**Authors:** Xiaole Chen, Kaimei Nie, Xin Zhang, Shuangyu Tan, Qingmei Zheng, Yaduan Wang, Xiaofeng Chen, Zhiyu Tang, Rui Liu, Mengru Yan, Zhiwei Liu, Jianbo Lin, Jianhua Xu, Nanwen Zhang, He Wang, Juhua Yang

**Affiliations:** 1 Department of Bioengineering and Biopharmaceutics, School of Pharmacy, Fujian Medical University, Fuzhou, Fujian, China; 2 Fujian Key Laboratory of Drug Target Discovery and Structural and Functional Research, Fujian Medical University, Fuzhou, Fujian, China; 3 Department of Neurorehabilitation, Rehabilitation Hospital Affiliated to Fujian University of Traditional Chinese Medicine, Fuzhou, Fujian, China; 4 Department of Thoracic Surgery, The First Affiliated Hospital of Fujian Medical University, Fuzhou, Fujian, China; 5 Department of Pharmacology, School of Pharmacy, Fujian Medical University, Fuzhou, Fujian, China; 6 School of Integrative Medicine, Fujian University of Traditional Chinese Medicine, Fuzhou, Fujian, China

**Keywords:** Autophagy, Inclusion bodies, Rheumatoid Arthritis, Tumor necrosis factor, VHH

## Abstract

The currently used anti-cytokine therapeutic antibodies cannot selectively neutralize pathogenic cytokine signaling that cause collateral damage to protective signaling cascades carrying the potential for unwanted side effects. The variable domains of heavy-chain only antibodies (HCAbs) discovered in Camelidae are stable and display to be fully functional in antigen-binding against variable targets, which seem to be attractive candidates for the next-generation biologic drug study. The purpose of our study was to establish a simple prokaryotic expression system for large-scale expression, purification, and refolding of the recombinant anti-tumor necrosis factor α (TNF-α) fusion protein (FVH1-1) from inclusion bodies. Over 95% purity of the recombinant anti-TNF-α fusion proteins was obtained by just one purification step in our developed prokaryotic expression system, while the results of surface plasmon resonance (SPR) established the high-efficiency potent binding ability of FVH1-1 to human TNF-α. The counteraction of TNF-α cytotoxic effect experiment on the mouse fibroblast fibrosarcoma cell line (L929) confirmed that the expressed FVH1-1 were able to selectively and highly combine with human recombinant TNF-α (hTNF-α) in *vitro*. Western blot results showed that FVH1-1 can inhibit the activation of caspase-9 and PARP, which are the apoptotic signaling pathway proteins activated by hTNF-α. Meanwhile, lysosome autophagy signaling pathways stimulated by hTNF-α were inhibited by FVH1-1, which down-regulated the expression of LC3II/LC3I and up-regulated the expression of P62, indicating that the autophagy linked with TNF-α-induced apoptosis in response to rheumatoid arthritis. The results of the AIA rat model experiment presented that FVH1-1 can reduce the degree of joint swelling and inflammatory factors to a certain extent in *vivo*.

## Introduction

Tumor necrosis factor α (TNF-α), a multifunctional immunomodulatory molecule secreted by stimulated mononuclear macrophages and some other cells *in vivo*, can bind to the cytomembrane receptor, leading to the local aggregation of immune effector cells or the death of target cells [1,2]. The activated inflammation signal transduction pathways by the proper stimulations result in the expression and release of inflammatory mediators including TNF-α, which is essential for the host defense. However, the excessive inflammatory responses lead to the pathological processes of multiple diseases, such as metabolic disease, autoimmune diseases, and chronic inflammation [3]. Therefore, avoiding either insufficient or excessive inflammatory responses strictly to keep the inflammation mediators balance regularly is extremely important in the innate immune system [4].

Anti-cytokine therapies and immune checkpoint inhibitors, representing antagonists or inhibitors of signaling cascades related to be pathogenic in a particular disease state, strongly improve the treatment of autoimmune diseases and cancer [5]. TNF-α acts as the most proximal mediator of the cytokine cascade among the inflammatory mediators, where serum TNF-α level can be increased within 1–2 h after the innate immune system with regard to the infections [2,6]. Thus, TNF-α has been widely accepted as an attractive target for biologic drugs against rheumatoid arthritis and other autoimmune diseases. Many currently used therapeutic antibodies can neutralize TNF-α and negatively regulate the activity of TNF-α *in vivo* [7]. However, when such anti-TNF-α antibodies are applied systemically, it is difficult for them to separate pathogenic signaling from physiological signaling, thus, leading to limited clinical efficacy and unwanted side effects [5]. Furthermore, most current clinically used therapies are expensive. A more cost-effective alternative anti-TNF-α therapeutic antibody that can target the related cognate antigens only in the particular organ or cell lineage is, therefore, urgently required.

The variable domain of heavy-chain only antibodies (HCAbs) first discovered in Camelidae, referred to as VHH or nanobodies (Nbs), can potentially address the problems of the anti-TNF-α therapeutic antibodies that are currently being used [8]. Apart from the unique characteristics of VHH, which include high stability and solubility, low immunogenicity, and excellent affinity to almost all possible targets, the particular aspect is that two or even three VHHs can be linked in a single polypeptide chain easily by utilizing the genetic engineering method to create bispecific reagents, which can target one or two pro-inflammatory cytokines, such as TNF or IL-6, at the same time [5,9]. Meanwhile, by binding the additional anti-cytokine moieties or modules directing to either specific organs or cell types in the VHH bifunctional fusion proteins, can not only increase the half-life of antibodies *in vivo*, but also selectively neutralize pathogenic cytokines while leaving normal function intact [10].

The VHH fragments have been reported to be expressed in prokaryotic systems or yeast, though in some cases, the transgenic protein levels were relatively low that require future improvements that are necessary. To overcome this limitation, the expression of the recombinant anti-TNF-α fusion proteins (FVH1-1), by linking three single domain chains, anti-TNF-α/HSA/TNF-α, was attempted in different conditions in *Escherichia coli* BL21 (DE3) cells. Our own studies simplified the engineering, expression, and purification of VHH technology. The present study aimed to explore the therapeutic effect of the recombinant anti-TNF-α fusion proteins (FVH1-1), obtained by our developed prokaryotic expression system, on adjuvant-induced arthritis (AIA) rats, and elucidated its underlying mechanism of autophagy action related to inflammatory mediator TNF-α in rheumatoid arthritis.

## Materials and methods

### Medium composition

The Luria–Bertani (LB) medium containing 1% tryptone, 1% NaCl, and 0.5% yeast extract (plus 1.5% agar in plates) was supplemented with appropriate antibiotic selection for the selection of transformants. The LB medium supplemented with 0.01 M MgCl_2_ and 0.02 M glucose was used as an electroporation medium.

### Strains and vectors

The pET30a+ vector, and *E. coli* BL21(DE3) strain (Beijing Quanshijin Biotechnology Company) were used for overexpression of the recombinant anti-TNF-α fusion proteins (FVH1-1).

### Construction of overexpression plasmids

The cDNA encoding the recombinant anti-TNF-α fusion proteins, FVH1-1, using the sequence information published in patent US2010/0172894 and further modified by software Codon usage database (http://www.kazusa.or.jp/codon), JCAT (http://www.jcat.de), and DNA work (http://mcl1.ncifcrf.gov/dnaworks), was made as a synthetic gene (Generay Biotech Co., Ltd) flanked by restriction sites for EcoRI and NotI. This was cloned by cohesive-end ligation into the multiple cloning sites of pET30a+, in frame with a plasmid 6× His tag at the gene 3′-end. The construct was verified by sequencing.

### Expression of the recombinant anti-TNF-α fusion proteins

The recombinant pET30a-6× His-anti-TNF-α fusion proteins plasmid was transformed into *E. coli* BL21(DE3) cells by the heat shock method for protein overexpression. The transformed *E. coli* BL21(DE3) cells were selected on LB agar plates containing 100 μg/ml ampicillin. One colony was picked and used to inoculate 5 ml LB medium containing 100 μg/ml ampicillin. The selected transformants were checked by PCR and digestion using restriction enzymes (EcoRI and NotI). The culture was grown at 37°C with 250 rpm, and was shaken until OD600 of 0.6 was achieved. IPTG (Sigma) was added to a final concentration of 1 mM to induce the expression of FVH1-1. After induction, the culture was shaken at 37°C for 4 h, and then harvested by centrifugation and stored at −20°C prior to protein purification.

### Isolation and refolding of the recombinant anti-TNF-α fusion proteins and purification under denaturing conditions

Transformed *E. coli* BL21(DE3) cells were harvested by centrifugation at 10000×***g*** for 5 min at 4°C. The wet weight of the cell pellet is noted and resuspended in 30 ml of lysis buffer (50 mM Tris-HCl, pH 7.9, 0.1 mM EDTA, 5% glycerol, 0.1 mM DTT, 0.1 M NaCl), following sonication for three to four intervals of 20 s with 1 min on ice. The entire lysate was centrifuged at 12000×***g*** for 10 min to separate the soluble and insoluble fractions. The pellet containing the inclusion bodies were resuspended in 30 ml of lysis buffer with 20 mM Tris buffer (pH 8.5) containing 1 mM EDTA, 1 mM reduced glutathione, put on ice for 10 min, and then centrifuged for 10000 rpm for 15 min. The inclusion bodies were dissolved into 100 mM Tris buffer (pH 12) with 2 M urea for 30 min at room temperature and then centrifuged at 15000 rpm for 10 min. The inclusion bodies were then washed by 3 M urea. The washed pellet containing proteins in the form of inclusion bodies was stored for further studies.

The FVH1-1 was affinity purified under denaturing conditions, using BeaverBeads™ IDA-Nickel with His-tag (Suzhou Beaver Bioengineering Company). The particles were washed with washing buffer containing 8 M urea, 100 mM NaH_2_PO_4_, 50 mM imidazole, and 10 mM Tris-HCl (pH 8.0). The denatured protein was then eluted with elution buffer containing 8 M urea, 100 mM NaH_2_PO_4_, 250 mM imidazole, and 10 mM Tris-HCl (pH 8.0), followed by desalination with Äkta (GE Healthcare, U.S.A.). The purity and yield of the protein were analyzed using 10% SDS/PAGE.

### Refolding of the recombinant anti-TNF-α fusion proteins

The solubilized protein was 20-fold diluted by freshly distilled deionized water. The protein aggregates were removed by centrifugation at 12000×***g*** at 4°C for 30 min, and the supernatant was recovered and carefully collected. SDS/PAGE and Western blot were performed to confirm the quality of recombinant protein expression.

### SDS/PAGE and Western blot

Prior to electrophoresis, samples were incubated at 95°C for 5 min in sample loading buffer (0.25 M Tris-HCl, pH 6.8, 5% glycerol, 5% 2-mercaptoethanol, 3% SDS, and 0.2 mg/ml Bromophenol Blue), and then separated by SDS/PAGE using 10% (w/v) acrylamide in the resolving gels. The samples were resolved on SDS/PAGE gels in an electrophoresis unit (Bio-Rad), 100 V, and constant current, for 1 h, using SDS running buffer (25 mM Tris, 200 mM glycine, 0.1% SDS, pH 8.3). The total proteins were detected in the gels using Coomassie Blue staining. For Western blots, the proteins separated by electrophoresis were transferred to the PVDF membrane and incubated overnight with an anti-His-tag mouse monoclonal antibody (Genetex, 1:2000) that was used to detect FVH1-1. The blot was washed three times with TBST. An HRP-labeled secondary antibody was then added and incubated for 1 h at room temperature. The blot was washed three times with TBST, reacted with ECL Western blotting reagents for 2 min, and then were exposed by Kodak Gel Logic Imaging Station for 15–60 s.

### Kinetic analysis of binding of the recombinant anti-TNF-α fusion proteins to TNF-α

Real-time binding interactions between ligand (biotinylated recombinant human TNF-α immobilized on a biosensor matrix) and analyst (antibodies in solution) were measured by surface plasmon resonance (SPR) using the biaCore system (GE Healthcare). This system utilizes the optical properties of SPR to detect alterations in protein concentrations within a dextran biosensor matrix. The recombinant anti-TNF-α fusion proteins are covalently bound to the dextran matrix at known concentrations. Human TNF-α antibodies are injected through the dextran matrix. Specific binding between injected antibodies and immobilized ligand results in an increased matrix protein concentration and results in change in the SPR signal. These changes in SPR signal are recorded as resonance units (RUs) and are displayed with respect to time along the y-axis of a sensorgram.

### Neutralization of TNF-α-induced cytotoxicity in L929 cells

Human recombinant TNF-α (hTNF-α) causes cell cytotoxicity to murine L929 cells after an incubation period of 18–24 h [11]. The recombinant anti-TNF-α fusion proteins were evaluated in L929 assays by co-incubation of antibodies with TNF-α and the cells as follows. A 96-well plate with clear flat bottoms (Costar) containing 100 μl of the recombinant anti-TNF-α fusion proteins was subjected to serial ten-fold dilution in duplicates using Dulbecco’s modified Eagle’s medium (DMEM) supplemented with 10% fetal bovine serum, penicillin (100 units/ml), and streptomycin sulfate (100 g/ml). Fifty microliters of hTNF-α was added for a final concentration of 500 pg/ml in each sample well. The plates were then incubated for 30 min at room temperature. Following, 50 μl of TNF-α-sensitive L929 cells were added for a final concentration of 5000 cells/well, including 1 μg/ml Actinomycin-D. Controls involved cells and hTNF-α. Plates were incubated for 24 h at 37°C in an atmosphere of 5% CO_2_ for further analysis. Cell viability was primarily determined using the 3-(4,5-dimethylthiazol-2-yl)-2,5-diphenyl tetrazolium bromide (MTT). Dose–response curves and 50% maximum response concentrations (EC_50_) were calculated with GraphPad Prism software (GraphPad Software, San Diego, CA).

The images of cell cytotoxicity were employed to further study the cell viability. L929 cells were seeded and treated with compounds as described above. After overnight incubation, cells were washed twice with PBS. After treatment, the cells were sequentially incubated with DAPI (10 μg/ml; Molecular Probes) and PI (10 μg/ml; Molecular Probes) for 10 min. The cells were washed three times with PBS for 10 min each time and were visualized using High-Content Analysis Instrument Platforms (ArrayScan, Thermo Fisher Scientific). Images of stained cells were acquired from the automated fluorescence microscope platform of the in ArrayScan using a 10× objective lens. Images from more than five fields per well were collected to obtain data on 200–400 cells. The filter sets, D360/40 excitation-HQ535/50 emission and D475/20 excitation-HQ 535/20 emission, were used for detection of DAPI and PI signals, respectively. The acquired images were analyzed using Cell Analyzer Workstation software according to the manufacturer’s instructions.

### Live lysosomal staining

Lysosome change was assessed with Lyso Green, a hydrophobic complex that can easily penetrate into living cells and selectively accumulate in lysosomes. It is widely used in studies regarding apoptosis, cytotoxicity, and cell viability. Briefly, L929 grown in 96-well plates were treated with hTNF-α for 24 h, with and without FVH1-1. Cells were then exposed to staining solution for 30 min in a humidified CO_2_ incubator, and directly examined under Leica fluorescence microscope.

### Immunoblot analysis

Cell lysates were prepared by scraping cells into an ice-cold buffer containing protease inhibitors and measuring the protein (BCA). The total proteins were separated by 12% SDS/PAGE and blotted on to PVDF membranes. Protein membranes were blocked with 5% milk and incubated with antibodies to LC3, P62 (Cell Signaling Technology) by overnight, followed by incubation with an HRP-conjugated anti-IgG secondary antibody (Millipore). The immunoreactive bands were detected by chemiluminescence. Densitometric analysis of the film images was performed with ImageJ software.

### Construction of the rat model of AIA

Eight rats were selected randomly from the 40 (SPF grade) as the control group, where each rat was intradermally injected with saline at 0.1 ml in the left hind paw pads. The other 32 rats were injected with an equal volume of Freund’s complete adjuvant (FCA) in the left hind paw pads to establish the AIA model, according to the method of Rice et al [12]. This was designated as day 0. The model animals were randomly assigned to four groups (*n*=8 in each group) on day 7: Model group; Recombinant Human TNF Receptor-Ig Fusion Protein for injection group, QiangKe, (AIA + 0.5 mg/kg per 3-day intravenous administration of QiangKe); high dose of the recombinant anti-TNF-α fusion protein group, FVH1-1, (AIA + 0.5 mg/kg per 3-day intravenous administration of FVH1-1) and low dose of the recombinant anti-TNF-α fusion protein group, FVH1-1 (AIA + 0.1 mg/kg per 3-day intravenous administration of FVH1-1). Simultaneously, the control and the model groups were intravenously treated with saline daily. Clinical evaluation was performed prior to immunization (baseline) and on alternate days following the initiation of drug treatment (post-dosing) up to 27 days, including standardized arthritis scores and measurements of edema [20]. After treatment, the rats were anesthetized by pentobarbital sodium. In the end, all the animals were killed by cervical dislocation. All procedures were performed in accordance with protocols approved by the Ethics Review Committee for Animal Experimentation of Fujian Medical University (No. 2017-052). All animals were raised in the Laboratory Animal Center of Fujian Medical University (Certificate No. SCXK (Fujian) 2016-0002), where the animal work took place. Animal handling procedures were performed in strict accordance with the care of laboratory animals according to the Fujian Province Zoological Society.

### Hematoxylin–Eosin staining

Kidney tissues were rinsed with ice-cold saline solution and fixed with paraformaldehyde (4%), embedded in paraffin, and cut into 3-μm sections. All sections were then stained with Hematoxylin–Eosin (HE) and photographed under an optical microscope with the magnification of 20×.

### Enzyme-linked immunosorbent assay

The levels of TNF-α, IL-6, and IL-1β were detected with commercial kits following the manufacturer’s instructions (enzyme-linked immunosorbent assay (ELISA) kits of TNF-α, IL-6, and IL-1β from Shanghai Enzyme Linked Biotechnology Co., Ltd, Shanghai, China).

### Statistical analysis

All data are expressed as means ± S.D. One-way ANOVA followed by Bonferroni’s Multiple Comparison Test using GraphPad software was used for comparisons among experiment groups. A *P*-value of less than 0.05 was considered statistically significant.

## Results

### Expression of the recombinant anti-TNF-α fusion proteins

**Figure 1:**
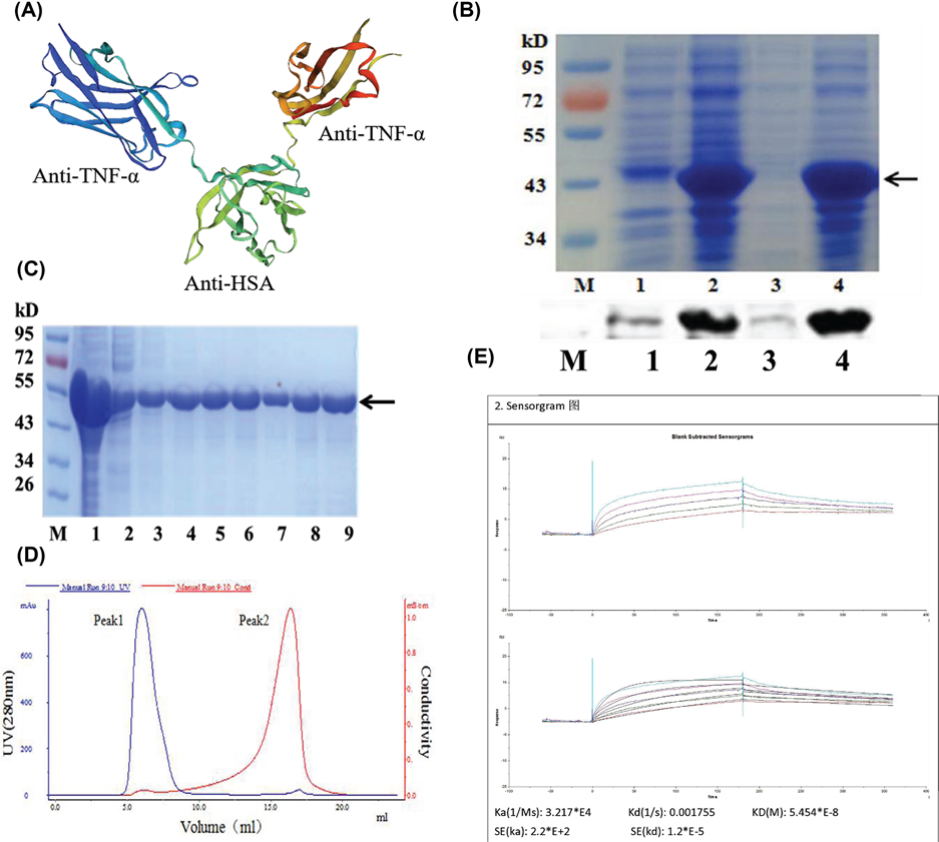
The preparation of the recombinant anti-TNF-α fusion protein, FVH1-1 (**A**) 3D structure of FVH1-1 mimicked by SWISS-MODEL. (**B**) Identification of FVH1-1 by SDS/PAGE and Western Blot. 1: uninduced, 2: IPTG-induced, 3: supernatant, 4: sediment, M: protein ladder. (**C**). Identification of the purity of FVH1-1 purified by IDA-Nickel with His-tag using SDS/PAGE. Coomassie Brilliant Blue staining 1: induced BL21(DE3)/FVH1-1; 2: sediment; 3: supernatant; 4–6: washed IBs; 7–8: renaturation protein; 9: further purified protein by IDA-Nickel. (**D**) Desalting with Äkta. (**E**) Kinetic analysis of binding of the recombinant anti-TNF-α fusion proteins to hTNF-α by BiaCore.

The cDNA encoding the recombinant anti-TNF-α fusion proteins, FVH1-1, was made synthetically based on the sequence information of patent US2010/0172894 (Figure 1A). The 39.27-kb inserts were ligated into the multiple cloning sites region downstream of the pET30a+ vector using the EcoRI/NotI restriction sites. The plasmids were transformed into the *E. coli* BL21(DE3) and selected on LB plates containing ampicillin. The selected transformants were confirmed by PCR and digestion using restriction enzymes (EcoRI and NotI).

The expression levels of the recombinant protein increased with time, reaching high levels of expression 4 h after induction. The presence of the recombinant protein in the induced *E. coli* cells was confirmed by a Western blot using His-tag specific antibody that allows visualization of His-tagged fusion proteins. As shown in Figure 1B, the recombinant anti-TNF-α fusion proteins were highly expressed in the form of insoluble inclusion bodies, with none in the soluble fraction. The inclusion bodies were purified by BeaverBeads™ IDA-Nickel. The process of purification was showed in Figure 1C. The recombinant anti-TNF-α fusion proteins sample was refolded at 4°C by using a step-wise approach, where the purified recombinant fusion protein was diluted by 20-fold freshly distilled deionized water. Desalting with Äkta was shown in Figure 1D. Based on SDS/PAGE, the purity of the fusion protein can achieve 95%. The KD of FVH1-1 against hTNF-α is 5.454 × 10^−8^ M determined by SPR exhibiting that FVH1-1 can bind to hTNF-α-related proteins specially (Figure 1E).

### Resistance to TNF-α-mediated killing of L929 cells by the recombinant anti-TNF-α fusion proteins

**Figure 2:**
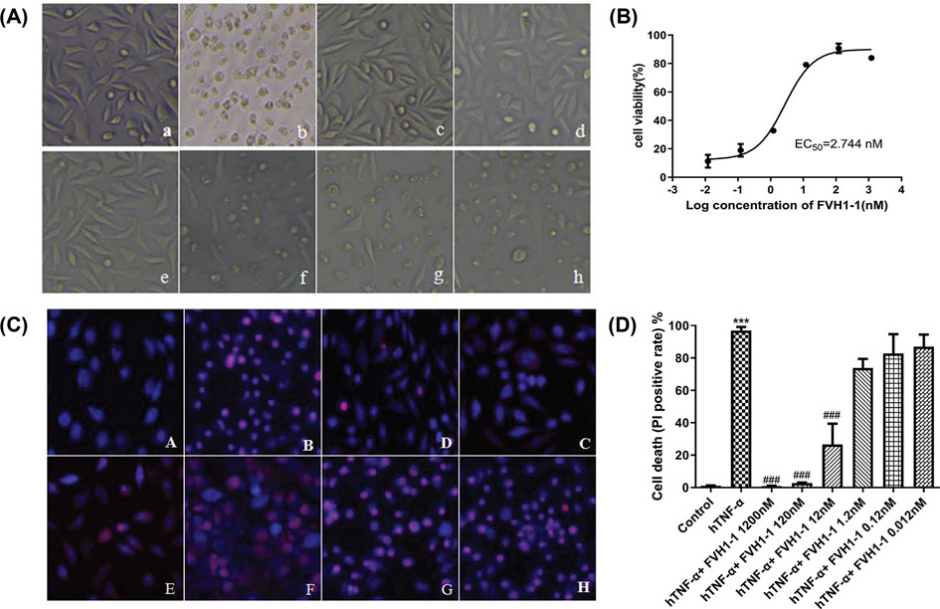
The inhibitory effects of FVH1-1 on the hTNF-α-induced L929 apoptosis (**A**) Morphological changes of L929 cells observed by upright microscope (10×): a. control, b. hTNF-α, c. hTNF-α+1200 nM FVH1-1, d. hTNF-α+120 nM FVH1-1, e. hTNF-α+12 nM FVH1-1, f. hTNF-α+1.2 nM FVH1-1, g. hTNF-α+0.12 nM FVH1-1, h. hTNF-α+0.012 nM FVH1-1. (**B**) The cells were cultured with 2 ng/ml hTNF-α and 10 μg/ml Actinomycin D in the presence of FVH1-1. The OD570 was determined by MTT assay. Error bars represent data from three independent. One-way ANOVA followed by Dunnett’s test was used for statistical analysis. (**C**) The cells were cultured with 2 ng/ml hTNF-α and 10 μg/ml Actinomycin D in the presence of FVH1-1 (1.2 μM, 0.12 μM, 12 nM, 1.2 nM, 0.12 nM, 0.012 nM) and then the cells were processed for DAPI/PI double stain assay. DAPI was used to stain the nuclei. PI was used to stain apoptotic cells. Error bars represent data from three independent experiments. ***P<0.0001 compared with the control group, ^###^*P*<0.0001compared with the hTNF-α group. Images were taken under fluorescent microscope. A. Negative control B. Positive control (2 ng/ml TNF-α), C. 1.2 μM FVH1-1+2 ng/ml hTNF-α, D. 0.12 μM FVH1-1+2 ng/ml hTNF-α, E. 0.012 μM FVH1-1+2 ng/ml hTNF-α, F. 1.2 nM FVH1-1+2 ng/ml hTNF-α, G. 0.12 nM FVH1-1+2 ng/ml hTNF-α, H. 0.012 nM FVH1-1+2 ng/ml hTNF-α. (**D**) High-Content Analysis of cell death ratio after PI staining.

The purified FVH1-1 exhibited dose-dependent inhibitory effects on hTNF-α induced cytotoxicity as low as 2.7 nM (Figure 2A,B). To visualize the effect of the recombinant anti-TNF-α fusion proteins on TNF-α-induced apoptosis, we used DAPI/PI staining to observe FVH1-1 protected from TNF-α-induced apoptosis of L929 cells. Under the fluorescence microscope, the negative control group showed uniform blue fluorescence, and the nuclei of the cells treated with TNF-α were uniformly colored by PI for a red color. The recombinant anti-TNF-α fusion proteins (FVH1-1) concentration of 1.2 μM, 120 nM, 12 nM can effectively reduce apoptosis in a dose-dependent manner (Figure 2C). High-content analysis of cell death results showed that the control group had an apoptotic rate of (1.00 ± 0.29)%, and the TNF-α (2 ng/ml) group had an apoptotic rate of (96.94 ± 2.23)%, which was significantly different from the control group (*P*<0.001). The apoptosis rates of FVH1-1 (1.2 μM, 120 nM, 12 nM) inhibiting the apoptosis of L929 induced by hTNF-α were 0.96 ± 0.13, 2.81 ± 0.33, and 26.53 ± 12.89%, which were significantly different from the 2 ng/ml hTNF-α-treated group (Figure 2D).

**Figure 3:**
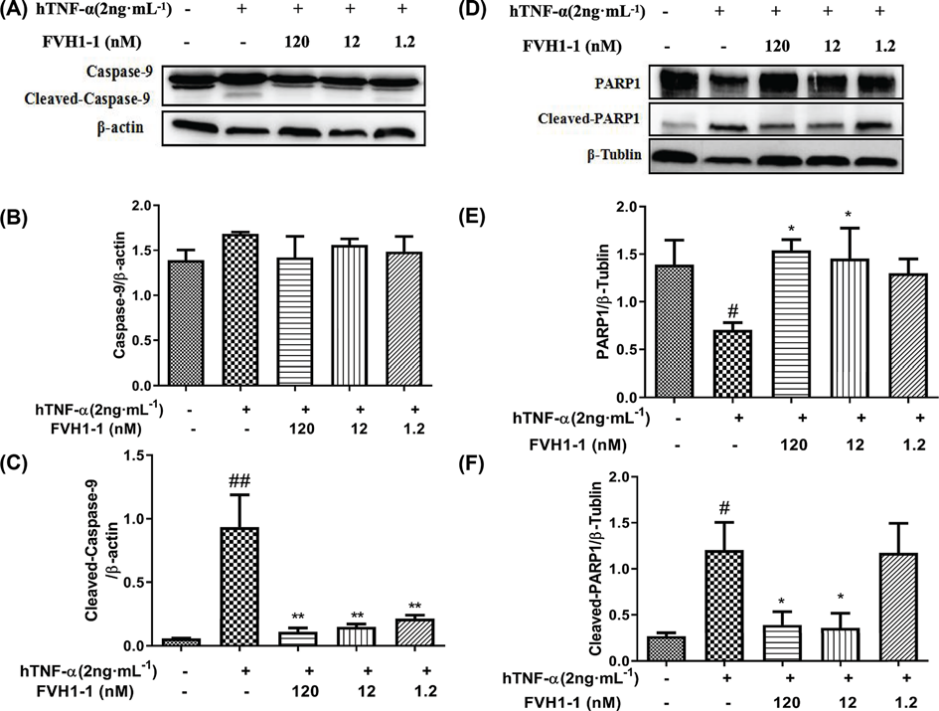
Effect of FVH1-1 on the expression of apoptosis-related proteins in L929 cells (**A**) Western blot analysis of the expression of Caspase-9 and Cleaved-Caspase-9 protein. (**B,C**) Caspase-9 and Cleaved-Caspase-9 gray value mapping, ^##^*P*<0.01 *vs* control, **P<0.01 *vs* hTNF-α. (**D**) Western blot analysis the expression of PARP1 and Cleaved-PARA1 protein. (**E,F**) PARP1 and Cleaved-PARA1 gray value mapping, ^#^*P*<0.05 *vs* control, *P<0.05 *vs* hTNF-α .

The Caspase family, playing critical roles in mediating apoptosis, exists in the form of inactive zymogen under normal circumstances. Once cells undergo apoptosis, Caspase can be cleaved by proteases, resulting in the formation of activated Caspase [13]. After treatment with hTNF-α (2 ng/ml) alone, the Caspase-9 (Figure 3A,B) and Caspase substrate PARP1, poly(ADP-ribose) polymerase (Figure 3D,E), of L929 cells were spliced and activated, where the expression of Cleaved-Caspase-9 (Figure 3C) and Cleaved-PARP1 (Figure 3F) were significantly decreased after the addition of different concentrations of FVH1-1, indicating that the recombinant TNF-α fusion protein can effectively inhibit TNF-α-mediated cleavage of Caspase-9 and PARP1 in L929 cells in a dose-dependent manner.

### The effects of the recombinant anti-TNF-α fusion proteins on hTNF-α induced autophagy-lysosomal during L929 cell apoptosis

**Figure 4:**
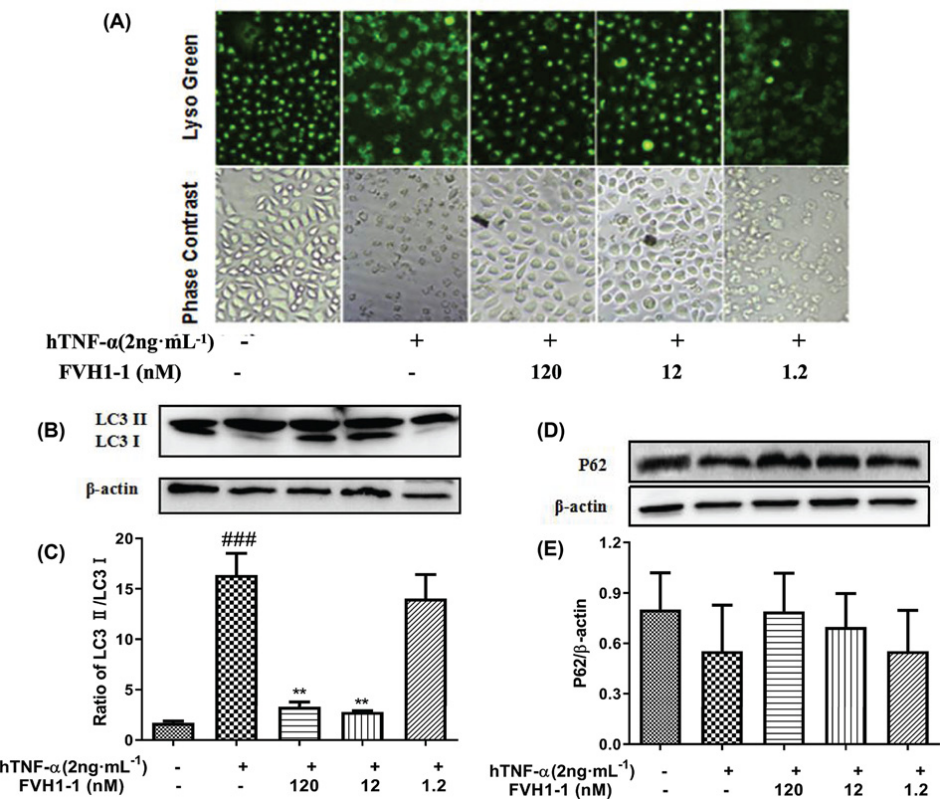
Effect of FVH1-1 on the hTNF-α-induced L929 autophagy (**A**) Effect of FVH1-1 on hTNF-α-mediated lysosomal staining in L929 necrosis. (**B**) The expression of lysosome-autophagy-related protein in L929 cells. The expression of LC3 (B) P62 (**D**) was determined by Western blot; Quantification of LC3II/LC3I (**C**) and P62 (**E**). ^###^*P*<0.001 *vs* control, **P<0.01 *vs* hTNF-α.

As shown in the Figure 4A, in the control group, the cells were evenly stained, and the green fluorescence was bright and apparent. The cells in the hTNF-α (2 ng/ml) treatment group shrank, dense staining appeared, and the green fluorescence was weak, where the fluorescence intensity was significantly lower than that of the control group, indicating that the cells were in poor condition and died. After treatment with different concentrations of FVH1-1, the morphology of FVH1-1 (120 nM, 12 nM) treatment groups was normal, and the green fluorescence intensity was significantly higher than that of the hTNF-α treatment group. The morphology and fluorescence intensity of FVH1-1 (1.2 nM) treatment group was close to hTNF-α treatment group. The results showed that lysosomes may also change during hTNF-α-mediated necrosis of L929 cells, and the addition of FVH1-1 inhibited the cytotoxicity induced by hTNF-α.

To further investigate whether hTNF-α mediates lysosomal autophagy during L929 cell apoptosis and the role of FVH1-1 in hTNF-α induced autophagy-lysosomal pathway, we detected the autophagy-associated protein LC3 and the substrate protein p62 [14] by Western blot. After treatment with hTNF-α (2 ng/ml) alone, the expression of LC3II protein was significantly increased, the LC3I protein was significantly attenuated, the conversion ability of LC3 from LC3I to LC3II was increased, the ratio of LC3II/LC3I was significantly higher than that of the control group (Figure 4B,C), and the expression of autophagy substrate P62 was decreased (Figure 4D,E). The results in Figure 4B–E exhibited that FVH1-1 could inhibit the lysosomal autophagy of L929 cells induced by TNF-α in a dose-dependent manner, demonstrating that FVH1-1 could effectively inhibit the autophagic death of L929 cells induced by hTNF-α.

### The effect of the recombinant anti-TNF-α fusion proteins on the inflammatory factors of the model foot in AIA rats

**Figure 5:**
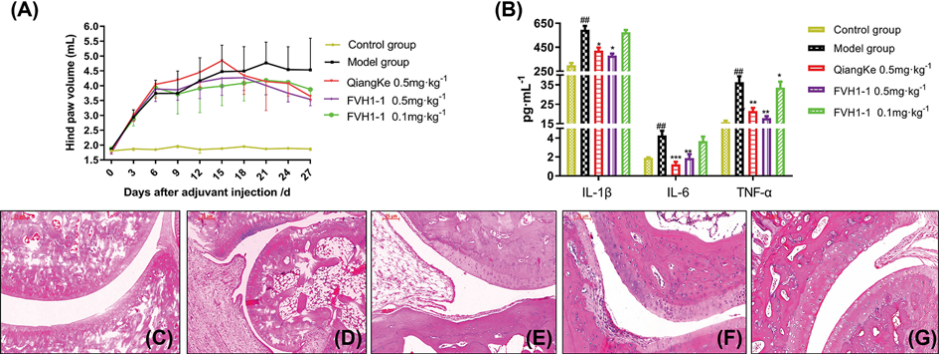
Effect of FVH1-1 on modelling foots inflammatory factors in AIA rats (**A**) Hind paw volume of WT and AIA rats treated with FVH1-1. (**B**) The expression of IL-6, TNF-α, and IL-1β in modeled feet, ^##^*P*<0.01 *vs* control group, *P<0.05, **P<0.01, ***P<0.001 *vs* model group. (**C**–**G**) Representative histological images of the posterior ankle joint in rats treated with FVH1-1.

After 1–6 days of CFA injection, the right hindfoot (modeling foot) of each group of rats showed obvious swelling, and the volume of the hindfoot and toes increased significantly, which can be regarded as successful adjuvant arthritis modeling. As shown in Figure 5A, after administration, the degree of swelling in the hindfoot volume of rats was significantly reduced (*P*<0.01).

The ELISA method was employed to detect the effects of the recombinant anti-TNF-α fusion protein, FVH1-1, on the inflammatory factors IL-6, IL-1β, and TNF-α in the modeled feet of AIA rats. As shown in Figure 5B, the expression level of IL-6, IL-1β, and TNF-α in modeling feet of model group was significantly increased with significant difference (*P*<0.001), compared with the control group. Whereas, the level of inflammatory cytokines IL-6 and TNF-α of Recombinant Human TNF Receptor-Ig Fusion Protein for injection group (QiangKe 0.5 mg/kg) and high dose of the recombinant anti-TNF-α fusion protein group (FVH1-1, 0.5 mg/kg) was significantly decreased with statistical significance (*P*<0.05, *P*<0.01 or *P*<0.001). The level of inflammatory factors of the low dose of fusion protein group (0.1 mg/kg) was slightly lower than that of the model group, but there was no statistical significance (*P*>0.05).

### Effect of the recombinant anti-TNF-α fusion protein on pathological HE staining of ankle joints in AIA rats

The ankle joints of rats were isolated for histological staining to observe the therapeutic effects of the recombinant anti-TNF-α fusion protein, FVH1-1. In the control group, the sacroiliac joint structure was intact, and the space between the joint cavities was normal; no synovial hyperplasia was seen, no synovial inflammatory cell infiltration was seen, and the articular cartilage structure was intact and clear (Figure 5C). In the model group, the structure of the sacroiliac joint was disordered, the space of the joint space was narrow, the synovial of the joint hyperplasia, synovial inflammatory cell infiltration, a lot of vasospasm formation, combined with cartilage and bone eroded (Figure 5D). Inflammatory cell infiltration and vasospasm formation were observed in Qiangke, the Recombinant Human TNF Receptor-Ig Fusion Protein, for the injection group (0.5 mg/kg) in Figure 5E. A small amount of inflammatory cell infiltration and synovial hyperplasia were observed in the high-dose FVH1-1 group (0.5 mg/kg) in Figure 5F, and the formation of vasospasm was significantly improved, while a large number of inflammatory cells were infiltrated in the low-dose group FVH1-1 (0.1 mg/kg), and the joint structure was incomplete and obvious (Figure 5G) .

## Discussion

TNF-α, as the proinflammatory cytokine, plays pathogenic roles in multiple diseases [15,16]. The therapeutic anti-TNF-α antibodies, including adalimumab, golimumab, infliximab, and certolizumab, are widely used in clinical treatment. However, such antibodies have one common problem that their discrimination of pathogenic signaling from physiological signaling is incomplete, causing collateral damage to beneficial or protective signaling cascades that carry unwanted side effects [5,7]. Meanwhile, expensive costs of current antibody therapies are inaccessible by most patients [17–19].

The ‘camel-specific’ heavy-chain antibodies lack both immunoglobulin light chains and the CH1 constant domain, though their only single variable domain (VHH) can recognize and bind to the antigen specifically [5]. Compared with conventional antibodies, VHHs have higher stability and are soluble, with smaller sizes exhibiting better penetration into tissues. Moreover, the simple form of VHHs can be employed to easily produce a recombinant protein that is able to target one or two antigens at the same time [20,21]. In this study, the recombinant anti-TNF-α fusion proteins (FVH1-1) allowing to target TNF-α and human serum albumin (HSA) was designed to bind to an abundant serum protein, leading to significant increase in the antibody’s half-life *in vivo*, as well as block cytokines at particular anatomical sites [10]. FVH1-1, encoding three anti-TNF-α/HSA/TNF-α VHH fragments, was successfully constructed by genetic engineering methods and was cloned in the pET30a+ expression vector, following being transformed into the *E. coli* BL21(DE3) strain for expression.

The bacteria BL21 (DE3)/pET30a-FVH1-1 was efficiently induced at 37°C and 1 mM IPTG for 5 h to express the recombinant proteins in the inclusion body at a high level. Notably, some paper reported that recombinant proteins were produced too rapidly to fold the nature structures in bacteria host cells, as well as the bacteria host cells lacking of most mammalian protein post-translational modifications, resulting in the induced proteins stored as inclusion bodies in an insoluble and inactive form. In these cases, the proteins cannot be expressed by *E. coli* [22–24]. However, the production of inclusion bodies was a welcome occurrence in our prokaryotic system, where overproduction of the recombinant protein FVH1-1 stored as inclusion bodies can be isolated with high purity by differential centrifugation and refolded with high efficiency. The inclusion body was washed by sodium deoxycholate (0.2%, 2%) and urea (2 M, 3 M), followed by being dissolved in 100 mM Tris-HCI (pH 12). SDS/PAGE electrophoresis and Coomassie Brilliant Blue staining showed that one step of affinity chromatography on IDA-Nickel resin can improve protein purity, and HiPrep™ 26/10 desalting can separate proteins from small molecule salt components such as urea.

L929 cells, sensitive to hTNF-α, were employed to explore the bio§functions of the recombinant anti-TNF-α fusion proteins (FVH1-1) that were obtained by our developed prokaryotic expression system *in vitro* [11]. Our results presented that FVH1-1 can neutralize hTNF-α cytotoxicity *in vitro* L929 assay with an EC_50_ of 2.744 nM. High content analysis results showed that the apoptosis rate of L929 apoptosis induced by 2 ng/ml hTNF-α was 96.94 ± 2.23%, and the apoptosis rates of FVH1-1 (1200 nM, 120 nM, 12 nM) inhibiting the apoptosis of L929 induced by hTNF-α were 0.96 ± 0.13, 2.81 ± 0.33, and 26.53 ± 12.89%, which were significantly different from the 2 ng/ml hTNF-α-treated. Western blot results showed that FVH1-1 can inhibit the activation of caspase-9 and PARP, which are the apoptotic signaling pathway proteins activated by hTNF-α [13].

It is interesting to find out that the recombinant anti-TNF-α fusion proteins (FVH1-1) displayed inhibition effects on lysosomal-autophagy mediated by hTNF-α during L929 cell apoptosis. As we know, autophagy is an important self-protective mechanism for cellular survival that plays critical roles in controlling the degradation of proteins and organelles. Basically, under non-stress conditions, the activity of autophagy is maintained at comparatively low levels. Once it is stimulated by cellular stressors, such as organelle damage and various infections, the autophagy activity can be strongly induced. Either excessive autophagic activity or inadequate autophagy can cause massive self-degradation or the accumulation of toxic materials resulting in the development of inflammatory disease, such as rheumatoid arthritis [25]. The live lysosomal staining experiment results exhibited that lysosomes changed during hTNF-α-mediated necrosis of L929 cells, while the hTNF-α neutralization by FVH1-1 can prevent this phenomenon. FVH1-1 can down-regulate the expression of LC3II/LC3I and up-regulate the expression of P62 of L929 stimulated by hTNF-α to inhibit lysosome-autophagy signaling pathways [14]. Recent studies have reported that suppression or deficiency of autophagy leads to the dysfunction and depletion of immune cells, followed by disturbed immunity under rheumatoid arthritis conditions, indicating that autophagy might be an effective therapeutic target for rheumatoid arthritis [3,4,25]. Study on the effects of hTNF-α on the lysosome autophagy can be useful for us to clarify the specific mechanisms that underlie inflammatory reprogramming and immunologic paralysis under rheumatoid arthritis.

The adjuvant-induced arthritis (AIA) rats, widely accepted as rheumatoid arthritis experimental model, were constructed to evaluate the bifunctions of the recombinant anti-TNF-α fusion proteins (FVH1-1) *in vivo*. The results showed that FVH1-1 can potently reduce the degree of joint swelling in the model, meanwhile inflammatory factors IL-6, IL-1β and TNF-α in the modeling feet were also significantly decreased by FVH1-1 with statistical significance in a dose manner. The ankle joints of rats that were isolated to make sections for histological staining presented that FVH1-1 can effectively inhibit the production of vasospasm in the arthritis model. Furthermore, compared with current clinically used anti-TNF-α antibodies, QiangKe (a recombinant Human TNF Receptor-Ig Fusion Protein), our recombinant anti-TNF-α fusion protein, FVH1-1 displayed the similar therapeutic effects on the treatment of rheumatoid arthritis in the rat model, and even showed stronger improvement at the same dose. Our yield and specific activity results definitively demonstrate that a simple prokaryotic expression system for large-scale expression, purification, and refolding of the recombinant anti-TNF-α fusion proteins (FVH1-1) from inclusion bodies has been established. Over 95% purity of the recombinant anti-TNF-α fusion proteins can be obtained from just one purification step in our developed prokaryotic expression system, which could be widely employed in the industrial production that can cut down massive production fees.

## Abbreviations

AIA: adjuvant-induced arthritis
DAPI: 2-(4-Amidinophenyl)-6-indolecarbamidine dihydrochloride
EC_50_: 50% maximum response concentration
ELISA: enzyme-linked immunosorbent assay
HE: Hematoxylin–Eosin
HRP: Horseradish Peroxidase
HSA: human serum albumin
hTNF-α: human recombinant TNF-α
IL: Interleukin
IPTG: Isopropyl-β-D-thiogalactoside
LB: Luria–Bertani
LC3: light chain 3
OD: optical density
PARP: poly(ADP-ribose) polymerase
PI: Propidium Iodide
SPF: Specific pathogen Free
SPR: surface plasmon resonance
TBST: Tris-HCl Buffer Solution -Tween
TNF-α: tumor necrosis factor α
